# The influence of speech stimuli contrast in cortical auditory evoked potentials

**DOI:** 10.5935/1808-8694.20130059

**Published:** 2015-10-04

**Authors:** Kátia de Freitas Alvarenga, Leticia Cristina Vicente, Raquel Caroline Ferreira Lopes, Rubem Abrão da Silva, Marcos Roberto Banhara, Andréa Cintra Lopes, Lilian Cássia Bornia Jacob-Corteletti

**Affiliations:** a PhD, Associate Professor - University of São Paulo; Associate Professor - Department of Speech and Hearing Therapy - School of Dentistry - University of São Paulo, Bauru campus, Brazil.; b Speech and Hearing Therapist; MSc student in Sciences of the Processes and Communication Disorders - School of Dentistry of Bauru; University of São Paulo, Bauru campus, Brazil.; c Speech and Hearing Therapist, Specialist in Family and Community Health - Federal University of São Carlos, São Carlos, São Paulo, Brazil).; d PhD; Professor. Speech and Hearing Therapist - Cochlear Implant and Cleft Lip and Palate Program - Santo Antônio Hospital- Irmã Dulce social works, Salvador, Bahia, Brazil.; e PhD, Associate Professor - University of São Paulo; Associate Professor - Department of Speech and Hearing Therapy - School of Dentistry - University of São Paulo, Bauru campus, Brazil. University of São Paulo.

**Keywords:** audiology, auditory pathways, electrophysiology, event-related potentials, P300, evoked potentials, auditory

## Abstract

Studies about cortical auditory evoked potentials using the speech stimuli in normal hearing individuals are important for understanding how the complexity of the stimulus influences the characteristics of the cortical potential generated.

**Objective:**

To characterize the cortical auditory evoked potential and the P_3_ auditory cognitive potential with the vocalic and consonantal contrast stimuli in normally hearing individuals.

**Method:**

31 individuals with no risk for hearing, neurologic and language alterations, in the age range between 7 and 30 years, participated in this study. The cortical auditory evoked potentials and the P_3_ auditory cognitive one were recorded in the Fz and Cz active channels using consonantal (/ba/-/da/) and vocalic (/i/-/a/) speech contrasts. Design: A cross-sectional prospective cohort study.

**Results:**

We found a statistically significant difference between the speech contrast used and the latencies of the N_2_ (*p* = 0.00) and P_3_ (*p* = 0.00) components, as well as between the active channel considered (Fz/Cz) and the P_3_ latency and amplitude values. These correlations did not occur for the exogenous components N_1_ and P_2_.

**Conclusion:**

The speech stimulus contrast, vocalic or consonantal, must be taken into account in the analysis of the cortical auditory evoked potential, N_2_ component, and auditory cognitive P_3_ potential.

## INTRODUCTION

The study of the P_3_ auditory cognitive evoked potential, enables the assessment of the neurophysiological cognitive processes which happen in the cerebral cortex, such as memory and auditory attention^1^. Since this is an objective method, its clinical applicability has been shown in different neurological and mental conditions, alterations in hearing, language, learning and others^2^, ^3^, ^4^, ^5^, ^6^.

Two auditory stimuli are utilized in the oddball paradigm, one rare and one that is frequent; they have a contrast between each other and are built based on frequency, intensity, meaning or category. Using two recording channels, it is possible to observe the N_1_, P_2_ e N_2_ cortical potentials for the frequent stimuli, and the P_3_component for the rare stimulus. The number used to name these components pertains to the order of occurrence in which these potentials are recorded, and the letters are used to characterize positive (P) and negative (N) peaks. It is important to stress that the P_3_ is considered a cognitive potential different from the others, since it corresponds to the electrical activity which happens in the auditory system when there is discrimination of the rare stimulus among the frequencies.

Studies have characterized the P_3_ component as to latency and amplitude as it is evoked by pure tones in individuals who can hear. However, the acoustic signal processing happens in a very different way vis-à-vis verbal and non-verbal sounds^7^, ^8^, ^9^, ^10^, and it is very difficult to generalize auditory processing information of a simple stimulus and a more complex one, like speech^11^.

The P_3_ cognitive auditory evoked potential generated by speech has been utilized to provide speech signal processing information when the behavioral assessment is not an accurate method, besides helping to pinpoint detection or discrimination alterations, and such information may guide the therapeutic rehabilitation of the individual^12^.

Thus, studies involving auditory evoked potentials with speech stimuli are important in order to understand how the stimulus complexity influences the characteristics of the potential generated, such as latency and amplitude. Table 1 depicts the latency values from the P_3_ cognitive and cortical auditory evoked potential latency values, as well as the amplitude values as evoked by speech (syllables) stimuli in adults with normal hearing.

**Table 1:** Mean values of the N_1_, P_2_, N_2_ e P_3_ component latencies (milliseconds) and amplitude values (*μ*V) from the P_3_ component in adults.

Study	N_1_	P_2_	N_2_	P_3_	P_3_ amp.
Sharma et al.^13^	117.0 (± 4)	-	-	-	-
Tampas et al.^14^	-	-	-	398.9	0.025
Gilley et al.^15^	108.0 (± 16)	176.0 (± 14)	-	-	-
Garinis & Cone-Wesson^16^	40 dBSL: 110 ms	40 dBSL: 200 ms	-	40 dBSL /sa/: 355 /da/: 345	5.67 (± 4.71)
Massa et al.^17^	-	-	-	348.95(± 29.69)	6.61(2.76)
Bennett et al.^18^	-	-	-	363(± 7.7)	4.7 (± 0.6)

amp.: amplitude.

The goal of the present paper was to characterize cortical auditory evoked potentials and the P_3_ cognitive auditory potentials from speech stimulus with vocalic and consonantal contrasts in normal hearing individuals.

## METHOD

This is a cross-sectional and prospective study carried out with the approval of the Ethics Committee, process # 069/2003. All the individuals assessed, or their guardians, signed the Informed Consent Form prior to being submitted to the exam.

We assessed 31 normal hearing individuals, without past disorders putting them in risk of developing auditory, neurological and language disorders, within the age range between 7 and 30 years, 13 females and 18 males.

The lack of hearing loss was proven by the auditory threshold of ≤ 25 dBHL upon threshold tonal audiometry, 92% scores for monosyllable words in the speech recognition index (SRI), type A tympanometry curve and acoustic reflex between 70 and 90 dBSL. We used the 622 Madsen audiometer^®^, with TDH-39 headphones, calibrated in the ANSI-69 standard and the Interacoustics AZ7^®^ immittance audiometer.

During the test, the individuals remained lying down in a gurney, in the dorsal position, and were instructed to keep their eyes as fixed as possible in order to reduce the artifact caused by eye movement. As we identified the rare stimulus among the frequent ones, the individuals were instructed to perform a simple motor action (raise the hand).

The simultaneous recording of the N_1_/P_2_ e N_2_/P_3_complexes in channels Fz and Cz was considered as a criterion to define the presence of cortical auditory evoked potentials and the P_3_ cognitive auditory potential. We used the Biologic's Evoked Potential System^®^ (EP) with the parameters described on Table 2.

**Table 2:** Parameters utilized in the study of cortical evoked potentials and the P_3_ cognitive auditory potential.

Assessment parameters	
Type of stimulus	Speech stimulus (80% frequent and 20% rare)
Stimulus frequency	Vowel contrast: /i/ (frequent); /a/ (rare) Consonant contrast: /ba/ (frequent); /da/ (rare)
Stimulus presentation rate	1 stimulus per second
Electrode positioning	Fz and Cz (active); A1/A2 (reference)
Pre-amplifer	Channels 1 and 2: input 1 - active electrodes; input 2 - reference electrodes (jumper)
Impedance	≤ 5 kΩ (individual); ≤ 2 kΩ (between electrodes)
Band-pass flter	1-25 Hz
Window	520 ms
Gain	75000
Intensity	70 dBHL, binaural stimulation
Transducer	3^rd^ insertion phone

The speech sample was collected in an acoustically treated room inside a lab. The emissions were recorded by means of a unidirectional microphone, directly on the computer board, through the Praat^®^ (www.praat.org) free software, with 22 kHz sampling. We asked the speaker (22 year-old male with a fluid voice quality) to utter the emissions naturally. In the beginning, we worked on the contrast by means of the /ba/-/da/ articulation point. By the spectral and temporal definition, the /ba/ was setup as a frequent stimulus, and the /da/ as the rare one. The [ba] and [da] syllables were taken from uttering the words [ba’ba] and [da’da], respectively, corresponding to the second syllable. From the isolated syllable, we found the F_1_, F_2_and F_3_ values in their initial and stable portions. With the bandwidth values of the forming frequencies stable regions we compiled a Praat script (version 4.2.31) and we resynthesized each syllable. The duration of the [ba] and [da] syllables was 180 ms. The /i/-/a/ meeting of vowels was established by the frequencies from formants F_1_ and F_2_ and by a shorter F_3_ extension. Vowels [a] and [i] were taken from the isolated utterance of syllables [pa] and [pi], respectively. In each syllable of the vowel region, we collected two glottic cycles with spectral stability, and in the Matlab^®^ (version 6.0.0.88), we replicated these cycles so as to correspond to the 150 ms vowel utterance. The vowels were created in the Praat^®^ with a script similar to what was previously described for the syllables. The linguistic stimuli which were previously produced, handled and recorded in a CD by the Lab were digitalized and inserted in the unit C of the computer connected to the software of the Biologic's Evoked Potential System^®^ (EP). The stimulus order and level of presentation were randomly handled by the aforementioned software.

In order to assess the results, we considered the absolute latency of the cortical auditory evoked potentials, N_1_, P_2_ and N_2_ components and P_3_ cognitive auditory, as well as the P_3_ component amplitude, obtained from channels Fz and Cz.

We compared the means among the types of channel and stimuli and the variable factors (amplitude and latency) utilizing a variance analysis model with repeated measures with two factors, ANOVA.

## RESULTS

**Figure 1:**
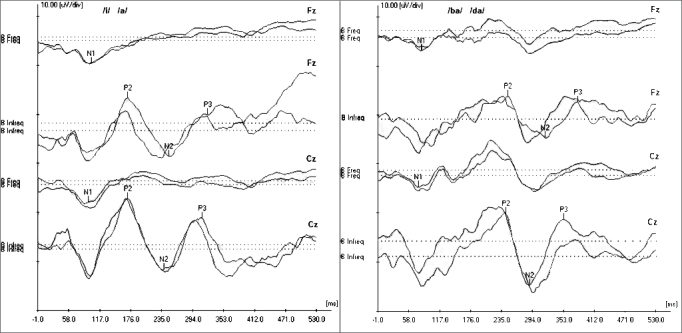
Record obtained in the study of the cortical auditory evoked potential and the P_3_ auditory evoked potential from a female individual with 29 years of age.

Figure 1 depicts an example of the recording obtained from studying the cortical auditory evoked potential and the P_3_ cognitive auditory potential in the Fz and Cz channels.

Upon investigating the occurrence of the records from N_1_, P_2_, N_2_ and P_3_ components, considering sample breaking down into the age ranges: 7-10 years; 11-20 years; 21-30 years, we can see the age influence on the recordings of components N_1_ and P_2_ (Table 3).

**Table 3:** Record occurrence (%) of components N_1_, P_2_, N_2_ and P_3_ considering the 7-10 years; 11-20 years and 21-30 years age ranges.

Age range (years)	N_1_	P_2_	N_2_	P_3_
7-10 (n = 9)	22.22%	66.66%	100%	77.77%
11-20 (n = 10)	90%	80%	100%	100%
21-30 (n = 12)	100%	100%	83.33%	100%

Table 4 depicts the descriptive analysis (mean, standard deviation, maximum and minimum values) of the N_1_, P_2_, N_2_ and P_3_ component latencies and P_3_ component amplitude, recorded from channels Fz and Cz, for all the individuals.

**Table 4:** Descriptive analysis (mean, standard deviation, maximum and minimum values) of the N_1_, P_2_, N_2_ e P_3_ component latency values in milliseconds and e P_3_ component amplitude (*μ*V) recorded in the Fz e Cz channels.

		Fz				Cz			
		X	SD	Minimum	Maximum	X	SD	Minimum	Maximum
N_1_	C	104	40	66	197	105	42	45	197
	V	106	17	75	139	103	33	50	170
P_2_	C	191	49	126	255	189	48	124	262
	V	186	35	117	240	179	36	99	230
N_2_	C	274	40	195	361	278	41	205	379
	V	236	38	153	289	239	27	182	278
P_3_	C	388	60	243	493	403	54	307	493
	V	322	39	226	376	339	44	249	447
P_3_ amp.	C	9	3	4	15	7	4	1	18
	V	10	5	2	23	7	3	2	14

X: Mean; SD: Standard deviation; amp.: Amplitude; C: Consonant; V: Vowel.

Our analysis of the association between the frequencies of components N_1_, P_2_, N_2_ and P_3_ and the P_3_component amplitude with the type of channel and the stimulus utilized did not show differences for the latency values of components N_1_ and P_2_. There was also a difference between the active channels (Fz and Cz) considered in the recording of the P_3_ component (Table 5).

**Table 5:** Study of the association between the channel type and stimulus factors and the N_1_, P_2_, N_2_ e P_3_ component latency variables and the P_3_ component amplitude.

Variation source	N_1_		P_2_		N_2_		P_3_		P_3_ amp.	
	F	*p*	F	*p*	F	*p*	F	*p*	F	*p*
Stimulus	0.11	0.74	1.10	0.30	16.26	< 0.01*	82.58	< 0.01*	0.01	0.90
Channel	0.04	0.82	0.99	0.33	0.47	0.49	10.95	< 0.01*	6.87	0.01*
Stimulus channel*	0.23	0.63	1.00	0.32	0.13	0.72	0.09	0.75	1.67	0.20

* Significant values (*p* ≤ 0.05) - ANOVA. amp.: Amplitude.

Table 6 depicts the Tukey Post-Hoc comparisons, considering the type of stimulus (consonant-vowel) for the latency of components N_2_ and P_3_ and considering the type of channel (Fz-Cz) for the amplitude and latency of the P_3_ component.

**Table 6:** List of the N_2_ e P_3_ component latency values considering the type of stimulus (consonant-vowel) and the amplitude and latency values of the P_3_ component with the Fz-Cz channel.

	Mean difference		Standard error	t	*p*	95% confidence interval	
	Stimulus	Channel				Lower limit	Upper limit
Amplitude P_3_	-	2.20	0.84	2.62	0.01*	0.47	3.94
Latency P_3_	-	-19.52	5.89	-3.31	0.01*	-31.63	-73.68
Latency N_2_	36.36	-	9.01	4.03	< 0.01*	17.61	55.11
Latency P_3_	66.86	-	7.35	9.08	< 0.01*	51.71	82.01

* Significant values (*p* ≤ 0.05) - Tukey's Post-Hoc comparisons.

## DISCUSSION

In the present investigation, it was possible to obtain the recordings of the cortical auditory evoked potentials and P_3_ cognitive auditory potential from a speech stimulus, with good reproducibility and morphology, showing that it is a viable procedure to be employed in clinical practice (Figure 1).

Analyzing the occurrence of recording from the N_1_and P_2_ exogenous components, it was possible to notice that their presence increased with age. The N_1_ component was practically nonexistent in the age range of 7-10 years corroborating the literature which states that, depending on the stimulus presentation characteristics, its recording can only be obtained as of 16 years of age, approximately^19^. Considering that the P_2_ component can also be influenced by the age range^20^, these data show the maturation process of the structures involved in the recording of the cortical auditory evoked potential.

Nonetheless, the age range did not influence the occurrence of recordings in N_2_ and P_3_ components, which are more frequently found than the N_1_ and P_2_components in children^21^. The gender variable was not analyzed, because in a study we did before we showed that there are no significant differences between males and females when we investigate the P_3_ auditory cognitive potential^22^.

In investigating the cortical auditory evoked potentials, we noticed that the N_1_ and P_2_ exogenous component latencies did not depict significant differences upon considering the Fz/Cz channel and the type of stimulus utilized (/a/-/i/; /ba/-/da/). Nevertheless, for the P_3_ cognitive auditory potential, the channel type was a factor which influenced its latency and amplitude, as per previously reported in other studies^22^, ^23^. By the same token, the type of stimulus used was an important variable in the attainment of N_2_ and P_3_ components.

The N_2_ component recording seems to be associated with the identification, processing and attention to the rare stimulus, with a positive correlation between the value of its latency and the level of difficulty in the discrimination task^24^. In our study, there was an influence of the speech stimulus on the N_2_ component, with higher latency values for the consonant contrast, suggesting that the degree of difficulty in the discrimination of such contrast is higher than the one found in the meeting of vowels. A similar finding was observed for the P_3_ component upon comparing verbal and non-verbal stimuli and in situations of difficult discrimination^14^, ^17^, ^18^, ^25^, reinforcing the hypothesis that this task is more difficult^26^.

However, this finding can also be explained by the evidence that vowels and consonants are processed in different ways by the central auditory system. One study carried out in rats^27^ compared discrimination behavioral responses from vowels and consonants with the neural recording from the inferior colliculus and primary auditory cortex, and suggested that consonants and vowels have different representations in the brain. In humans, studies have also reported differences in the activation of central auditory system structures during the discrimination of vowels and consonants^28^, ^29^. Therefore, the type of speech contrast used may reflect differently on the latency of the N_2_ and P_3_ components.

Some studies describe the reduction in the P_3_ component amplitude with the increase in the task's level of discrimination difficulty^14^, ^17^, ^18^, ^25^, ^26^. Nonetheless, this correlation was not significant in the present study.

In our series, the normal latency values for the N_1_, P_2_, N_2_ and P_3_ components for the vowel and consonant contrasts are depicted on Table 4. The comparative discussion between the values found and results from previous studies is inaccurate, because the methodologies are different, and as per shown above, assessment parameters such as type of stimulus utilized, have a significant influence on the latency values of auditory evoked potentials.

Considering that different neural structures are activated during the perception of verbal and non-verbal sounds, we stress the importance of using speech stimuli in future studies with the cortical auditory evoked potentials and the P_3_ cognitive auditory potential.

## CONCLUSION

The consonant or vowel-related speech stimulus, must be considered in the analysis of the N_2_ component of the cortical auditory evoked potentials and the P_3_ cognitive auditory potential. This was not observed for the N_1_ and P_2_ components.
